# Phytochemical Profiling and Antioxidant Potential of *Bongardia chrysogonum* Seed Essential Oil as a Natural Antioxidant: Microencapsulation With Sodium Caseinate and β‐Cyclodextrin Inclusion Complex

**DOI:** 10.1002/fsn3.71085

**Published:** 2025-11-05

**Authors:** Mohamad Mehdi Nematshahi, Nafiseh Nemat Shahi, Rozhan Sanavi Kordestani

**Affiliations:** ^1^ Department of Food Science and Engineering Islamic Azad University Hamedan Iran; ^2^ Department of Biology, Faculty of Science Ferdowsi University of Mashhad Mashhad Iran; ^3^ Department of Food Science and Engineering Islamic Azad University Mashhad Iran

**Keywords:** *Bongardia chrysogonum* seed essential oil, encapsulation, morphology, natural antioxidants, oxidative induction time

## Abstract

This study explored the phytochemical composition and antioxidant capacity of *Bongardia chrysogonum* seed essential oil (BCEO), reporting its properties and microencapsulation for the first time. GC–MS showed that BCEO was rich in unsaturated fatty acids (76.6%), with linoleic acid (49.4%) and oleic acid (25.2%) as major unsaturated and saturated fatty acids, respectively. HPLC analyses revealed high concentrations of γ‐tocotrienols (794.29 μg/g), α‐tocotrienols (635.88 μg/g), α‐tocopherol (540.85 μg/g), and phenolics such as quercetin, luteolin, and rutin. The total phenolic content reached 34.75 mg GAE/100 g, and the oxidizability value (COX) was calculated as 5.58 au. The incorporation of 1000 ppm of BCEO into butter increased oxidative induction time from 2.84 to 8.82 h, comparable to synthetic antioxidant BHA (9.33 h). BCEO was encapsulated via freeze‐drying using sodium caseinate (SC), β‐cyclodextrin (β‐CD), and their composite (β‐CD–SC). Microparticles were characterized for morphology, chemical structure, entrapment efficiency (EE%), moisture content, particle distribution index, and DPPH radical scavenging activity. The β‐CD–SC system exhibited the highest EE (88.48%) and DPPH activity (87.59%). SEM and FTIR analyses confirmed structural integration of oil into wall materials, with β‐CD particles showing the smallest size and the most agglomeration. The composite particles offered favorable moisture levels, narrow size distribution (PDI = 0.253) with particle sizes ranging from 122.4 nm to 295.3 nm (mean particle size of 206.4 nm), and potential for application in functional foods. These findings highlight BCEO as a promising natural antioxidant and support its stabilization through encapsulation in β‐CD–SC inclusion complexes.

## Graphical abstract

First‐time chemical profiling of *Bongardia chrysogonum* seed essential oil (BCEO). BCEO exhibited oxidative stability comparable to synthetic antioxidants. Encapsulated BCEO shows promise as a natural food preservative.

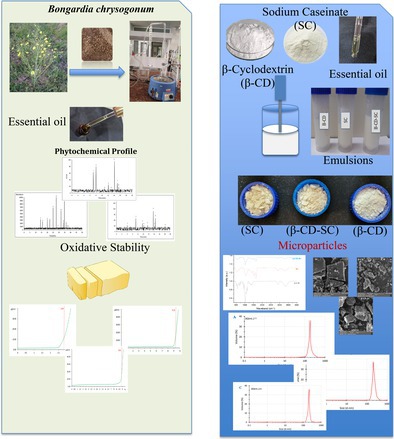

## 1 Introduction

Plants are valuable sources of natural secondary metabolites that have a wide range of bioactive compounds in food, pharmaceutical, and medical fields. Consequently, much recent research has focused on identifying novel and value‐added bioactive compounds from plants, particularly wild species (Al‐Bukhaiti et al. 2018). The genus *Bongardia L*. belongs to the Berberidaceae family, is distributed across the Eastern Mediterranean region, North America, and western Asia, including Iran, Turkey, Iraq, and Afghanistan (Gezici and Şekeroğlu 2021). Among its species, *Bongardia chrysogonum* is considered the most important species of the genus in the flora of Iran, which is locally known as “Alaf‐Kabki”. Khorasan, Kurdistan, and Lorestan provinces are three centers of variety for the genus (Freitag 1977; Pieroni et al. 2017). Previous studies on *B. chrysogonum* have primarily focused on extracts from its leaves, stems, and tubers, revealing notable antioxidant, antimicrobial, anticancer, and neuroprotective properties (Abuhamdah et al. 2017; Dokuyucu et al. 2016; Görmez 2024; Yousef et al. 2021). However, no prior research has investigated the chemical composition and antioxidant potential of *B. chrysogonum* seed oil. Meanwhile, fruit seed oils (e.g., grape, berry, avocado, and pomegranate seeds), given the growing interest in their health‐promoting components, including polyunsaturated fatty acids (PUFAs), phenolic compounds, and tocopherols, are increasingly utilized in functional foods and cosmetics (Alves et al. 2021; Górska et al. 2023; Setyawan et al. 2021; Yang et al. 2021). In this regard, the oil of the Berberidaceae family has become an interesting trend in recent research, revealing great bioactivity for such oils (Chorshanbiev and Berdiev 2022; Gıdık 2021; Górska et al. 2023; Iskender et al. 2024).

Essential oils are rich in PUFA and various bioactive compounds. Therefore, they are highly sensitive to environmental stresses, limiting their industrial application (Abbasi et al. 2019). Encapsulation, which forms a polymeric cover around the essential oil, can protect it against harsh environmental factors and improve its water solubility and bioavailability (Fu et al. 2024; Khoshnoudi‐Nia et al. 2022). Freeze drying is one of the most popular methods for encapsulating heat‐sensitive bioactive materials, as it removes the solvent via sublimation of ice crystals (Jafari et al. 2016).

The selection of wall material is critical in the encapsulation process and affects the encapsulation efficiency and stability of the encapsulated essential oil. Previous research has revealed that the protein–polysaccharide complexes can offer better encapsulation performance due to their synergistic effects in improving the stability and delivery of bioactive compounds (Cheong et al. 2018; Devecioglu et al. 2025; Jamshidian and Rafe 2024; Khoshnoudi‐Nia et al. 2022; Sharif et al. 2020). Among various protein sources, milk proteins are natural amphiphilic biopolymers, which contain both hydrophobic and hydrophilic groups, enabling excellent emulsifying and encapsulation properties (Khoshnoudi‐Nia et al. 2022). Sodium caseinate (SC) is a water‐soluble milk protein; its amphiphilic structure allows it to rapidly adsorb onto the oil/water interface and effectively decrease the interfacial tension, and thus stabilize the oil droplets in the aqueous phase (Cheong et al. 2018; Fenyvesi et al. 2016; Liao et al. 2022).

On the other hand, beta cyclodextrin (β‐CD) comprises seven D‐glucose units that are connected by α‐1,4 linkages (Escobar‐Avello et al. 2021). β‐CD forms an inclusion complex with hydrophobic compounds through weak forces, such as van der Waals force, dipole–dipole interaction, and hydrogen bonding, entrapping bioactive compounds inside its cavity. This unique structure enhances the solubility and stability of hydrophobic core materials, such as essential oils (Fu et al. 2024). Therefore, the combination of β‐cyclodextrin and sodium caseinate (β‐CD‐SC) can provide both inclusion complexation and interfacial stabilization, making it a promising encapsulation system, especially for hydrophobic compounds. Although β‐CD and/or SC have been successfully used to encapsulate several essential oils (Chew et al. 2018; Fu et al. 2024; Maraulo et al. 2024). To the best of our knowledge, no previous studies have reported the use of a β‐CD and SC combination as a wall material for freeze‐dried microparticles of fruit seed oils, including *Bongardia chrysogonum* seed essential oil (BCEO), highlighting the novelty of the present study.

Therefore, this study aims to (i) extract *B. chrysogonum* seed essential oil and analyze its phytochemical profile (free fatty acid profile, tocopherols, and phenolic compounds) for the first time; (ii) evaluate the essential oil's antioxidant activity and its impact on the oxidative stability of butter; (iii) encapsulate the essential oil using β‐cyclodextrin (β‐CD) and sodium caseinate (SC), and characterize the physicochemical properties besides the chemical and morphological structure of the microparticles.

## 2 Materials and Methods

### 2.1 Materials

The seeds of *Bongardia chrysogonum* (BC) were sourced from a local market in Neyshabur, Khorasan Razavi, Iran. Folin‐Ciocalteu phenol reagent (quality level of 200; density: 1.24 g/cm^3^ at 20°C) and 1,1‐diphenyl‐2‐picrylhydrazyl (DPPH, CAS‐Number: 1898‐66‐4; Molecular weight: 394.32 g/mol) were purchased from Sigma‐Aldrich (Sigma Chemicals Co, St. Louis, MO, USA). All other analytical‐grade chemicals and solvents, including gallic acid (ACS reagent grade, ≥ 98.0%), sodium carbonate (Na_2_CO_3_, ACS reagent grade ≥ 99.5%), methanol (HPLC grade, ACS reagent grade, ≥ 98.0%, ≥ 99.9%), and ethanol (purity = 96%) were obtained from Merck (Darmstadt, Germany), Sigma‐Aldrich (St. Louis, MO, USA), and Dr. Mojallali Chemical Complex Co. (Tehran, Iran).

### 2.2 Essential Oil Extraction Process

The seeds of *B. chrysogonum* were ground using a grinder (Vitamax, Pars‐Khazar, Iran) and passed through a sieve with a mesh size of 40. The essential oil of *B. chrysogonum* (BCEO) was extracted by hydro‐distillation for 3 h using a Clevenger‐type apparatus in a seed powder to water ratio of 1:10 v/w, and the yield of essential oil was 0.98% ± 0.08%. The obtained essential oil was dried over anhydrous sodium sulfate (Na_2_SO_4_) and stored in dark glass vials at 4°C until further analysis (Zoubiri and Baaliouamer 2010).

### 2.3 Identification of Free Fatty Acids Profile

The fatty acid composition of BCEO was analyzed using Gas Chromatography–Mass Spectrometry (GC–MS; Agilent 7890 Agilent Technologies, USA). The analysis was conducted under the following conditions: Injection volume of 1 μL, a capillary column measuring 60 m in length with an internal diameter of 0.25 mm, and helium as the carrier gas at a constant flow rate of 2 mL/min. The oven temperature was initially set at 40°C for 2 min, then programmed to rise to 150°C at a rate of 5°C/min, followed by an increase to 300°C at 15°C/min. The injector temperature was maintained at 280°C. The mass spectrometer operated with a scan range of 30–800 amu and an ionization voltage of 70 eV (Hu et al. 2005). Fatty acid identification was achieved by comparing the retention times and mass spectra of sample peaks with those of authentic standards. Data acquisition and processing were performed using Agilent MSD Chemstation software.

### 2.4 Identification of Tocols

The tocol composition of BCEO was evaluated by High‐Performance Liquid Chromatography (HPLC; Agilent Technologies 1200 series, Germany) equipped with a UV‐diode array detector (UV‐DAD) and according to the AOCS method Ce 8‐89. For sample preparation, 2 g of BCEO was diluted in 100 mL of hexane and subsequently filtered through a 0.45 μm nylon syringe filter. Tocols were eluted using an isocratic mobile phase composed of methanol, acetonitrile, and isopropanol, delivered at a flow rate of 1 mL/min. Tocols were detected at a wavelength of 290 nm. The identification of tocopherols and tocotrienols was done based on calibration curves generated from known standards, as milligrams per kilogram of oil (mg/kg) (AOCS 2024).

### 2.5 Identification of Phenolic Compounds

Phenolic compounds of BCEO were identified using HPLC analysis coupled with a UV‐DAD. The chromatographic conditions included an injection volume of 20 μL, a mobile phase flow rate of 1 mL/min, and a total run time of 40 min. Detection was carried out at 280 nm. Instrument control and data acquisition were conducted using ChemStation software (Waldbronn, Germany; Gull et al. 2018).

### 2.6 Total Phenolic Content (TPC)

The total phenolic content of BCEO was determined via the Folin and Ciocalteu method. Absorbance measurements were obtained at 750 nm using a UV–visible spectrophotometer (UV‐9200; Beijing Rayleigh Analytical Instrument Co., Beijing, China). The results were reported as milligrams of gallic acid equivalent per 100 g of essential oil (mg GAE/100 g; Atanassova et al. 2011).

### 2.7 Oxidizability Value (COX)

To assess the oxidative susceptibility of BCEO based on its fatty acid composition, the Oxidizability value (COX), a dimensionless parameter, was calculated using the following equation (Equation 1; Symoniuk et al. 2022):

(1) $$\mathrm{COX}=\frac{1\times \left(C16:1+C17:1+C18:1+C20:1\right)+10.3\times \left(18:2\right)+21.6\times \left(C18:3+C20:3\right)}{100.}$$

### 2.8 Oxidative Stability

To assess the oxidative stability of butter oil samples containing antioxidants (BCEO at concentrations of 0, 250, 500, and 1000 ppm and Butylated Hydroxyanisole (BHA) at 100 ppm), the Rancimat 733‐5 device (Metrohm, Herisau, Switzerland) was employed. Measurements of induction time (IT, expressed in hours) were carried out following the guidelines of the AOCS official method Cd 12b‐92. Each analysis used 2.5 g of oil under a constant airflow of 20 L/h at controlled temperatures ranging between 90°C and 130°C (AOCS 2000).

### 2.9 Preparation of Essential Oil Emulsions

In this study, three types of wall materials were employed: β‐cyclodextrin (β‐CD), sodium caseinate (SC), and a 1:1 (w/w) combination of the two (β‐CD‐SC). To prepare the encapsulating matrix, 20 g of each biopolymer was dissolved in 100 mL of distilled water under continuous agitation at 600 rpm and a controlled temperature of 35°C using a magnetic stirrer (RT 5, IKA‐Werke, Staufen im Breisgau, Germany). The solutions were left to hydrate overnight at ambient temperature with constant mechanical stirring to ensure complete hydration. Then, BCEO (EO to wall material of 1:4 (w/w)) and Tween 20 (as an emulsifier at 5% w/w of BCEO) were added dropwise to the wall material solution under magnetic stirring. Tween 20 plays a critical role in reducing interfacial tension and enhancing the stability of the oil‐in‐water emulsion (Gorjian et al. 2022). The mixture was homogenized at 10,000 rpm for 5 min at ambient temperature (25°C ± 2°C) using an Ultra‐Turrax homogenizer (IKA T25 Disperser Ultra‐Turrax, Staufen, Germany) to form a stable emulsion. The wall materials and their ratios were selected based on a literature review and initial screening conducted in the laboratory (data not shown; Chew et al. 2018; Kringel et al. 2021; Magri et al. 2025; Repajić et al. 2024).

### 2.10 Encapsulation of Essential Oil via Freeze‐Drying

Freshly prepared emulsions were first frozen at −80°C using a −80°C freezer (Jal Tajhiz, Iran) and then subjected to lyophilization using a freeze dryer (LD Plus4‐2 Alpha, Christ, Germany) operating at −30°C for 48 h. The freeze‐dried samples were passed through a 40‐mesh sieve. All powders were produced in duplicate.

### 2.11 Fourier Transforms Infrared Spectroscopy (FTIR)

The spectra of the encapsulated samples were analyzed using Fourier Transform Infrared (FTIR) Spectroscopy (IR Affinity, Shimadzu, Japan). The samples were homogenized with KBr at a typical sample‐to‐KBr ratio of 1:100 prior to analysis. Spectral data were collected across a wavenumber range of 4000–400 cm^−1^, at a resolution of 4 cm^−1^, with a scan speed of 2 mm/s.

### 2.12 Scanning Electron Microscopy Analysis

The surface morphology of freeze‐dried samples was examined with a scanning electron microscope (SEM) using a Tesca‐Vega3 instrument (Tescan Co., Czech Republic). Prior to imaging, samples were sputter‐coated with a thin gold layer approximately 10 nm thick. SEM analysis was conducted at an accelerating voltage of 20 kV, with images captured at a magnification of 1.00 Kx.

### 2.13 Encapsulation Entrapment (EE)

Encapsulation entrapment (EE) refers to the percentage of essential oil successfully entrapped within the sodium caseinate and/or β‐cyclodextrin. To determine the surface oil of microparticles, 1.0 g of each powder was rinsed with 10 mL of n‐hexane for 2 min at room temperature, followed by filtration through Whatman No. 1 filter paper. The retained powder on the filter was then washed three times with 20 mL of n‐hexane. The solvent was evaporated to a constant weight, and the resulting powder was weighed to determine the surface oil content. The total oil content was assessed by dispersing 1.5 g of encapsulated powder in 15 mL of distilled water and sonicated for 5 min at room temperature. Then, 15 mL of a 3:1 (v/v) n‐hexane:isopropanol mixture was added, followed by vortexing and centrifugation at 5000 rpm for 5 min. The isopropanol/water phase was carefully removed, and the oil phase was added to that from the surface oil extraction. The solvent was evaporated, and the total oil was weighed (Magri et al. 2025). EE% was calculated according to Equation (2):

(2) $$\mathrm{EE}\;\left(\%\right)=\frac{\text{total oil}-\text{surface oil}}{\text{total oil}\;}\times 100$$

### 2.14 Antioxidant Activity

The antioxidant potential of encapsulated samples was determined using the DPPH^●^ (2,2, diphenyl‐1‐picrylhydrazyl) free radical scavenging assay as described by Koleva et al. (2002). The absorbance of the reaction mixture was measured at 515 nm against a blank, and the radical scavenging activity was expressed as a percentage.

### 2.15 Moisture Content

The moisture content of the various microparticle formulations was determined by drying the samples in a vacuum oven at 105°C until a constant weight was achieved. The final moisture content was calculated and expressed as a percentage of the initial sample weight (Chen 2003).

### 2.16 Bulk Density

Bulk density was measured by transferring a known mass of the microparticle powder into a 10 mL graduated cylinder without tapping or compaction. The bulk density (g/cm^3^) was calculated by dividing the weight of the sample by the occupied volume (Abdullah and Geldart 1999).

### 2.17 Particle Size

The particle size distribution of the encapsulated essential oil powders was determined using laser diffraction with a Malvern Mastersizer particle size analyzer (Worcestershire, UK) equipped with a Scirocco 2000 dry dispersion unit (Repajić et al. 2024).

### 2.18 Statistical Analysis

Statistical evaluations were performed using the General Linear Model (GLM) procedure of analysis of variance (ANOVA). Significant differences between the means were assessed by Tukey's Honest Significant Difference (HSD) test at a 95% confidence level (*p* ≤ 0.05). All experiments were conducted in triplicate using individual samples, and results were expressed as mean values ± standard deviation (SD). Data processing and analysis were carried out using Minitab software (v20, Minitab Inc., Pennsylvania, USA).

## 3 Results and Discussion

### 3.1 Free Fatty Acids Profile

The *Bongardia chrysogonum* essential oil mainly consists of unsaturated fatty acids (76.6%). Previous findings also suggest that the Burseraceae family could be a valuable source of unsaturated fatty acids (Mazzuca et al. 2005; Nova‐Baza et al. 2022; Atefe Tavakoli et al. 2016). Also, grape seed oil has a high content (90%) of unsaturated fatty acids, especially linoleic acid (70%) (Matthäus 2008).

According to Table 1, palmitic acid (15.82%) was identified as the major saturated fatty acid, while linoleic acid (49.40%) was the predominant unsaturated fatty acid in BCDO. Oleic acid (25.2%) and stearic acid (4.37%) were other important fatty acids in this essential oil. Chorshanbiev and Berdiev (2022) also indicated that unsaturated fatty acids dominate barberry seed oil, with the combined proportion of ω‐9 oleic acid and ω‐3 linolenic acid accounting for 52% of the total free fatty acid profile, while ω‐6 linoleic acid accounted for 37.02%.

**TABLE 1:** Profiles of free fatty acids of *Bongardia chrysogonum* seed essential oil.

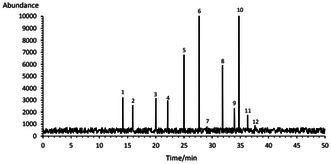

Peak #	Chemical	RT (min)	Concentration (%w/w)
1	Myristic Acid (C14:0)	25.01	0.96
2	Palmitic Acid (C16:0)	27.71	15.82
3	Palmitoleic Acid (C16:1)	29.08	0.92
4	Stearic Acid (C18:0)	31.85	4.38
5	Trans‐ Oleic Acid (t9C18:1+ t11C18:1)	33.95	3.01
6	**Cis‐ Oleic Acid (c9C18:1 + c6C18:1)**	**34.75**	**22.22**
7	**Linoleic acid (C18:2)**	**36.30**	**49.41**
8	Arachidic acid (C20:0)	37.02	1.20
9	Cis‐ Linolenic acid (c6, c9, c12C18:3)	37.60	1.07
10	Docosanoic acid (C22:0)	40.9	1.02

*Note:* The bold rows indicate the main components of the essential oil.

Abbreviation: RT, retention time.

Similarly, studies on the fatty acid profile of seeds from four wild species of the Berberidaceae family growing in Turkey showed that α‐linolenic acid (32.85%–37.88%), linoleic acid (30.98%–34.28%), and oleic acid (12.85%–19.56%) were the most abundant fatty acids. Palmitic acid (5.13%–6.35%) was also the most prevalent saturated fatty acid in seeds of the Berberidaceae family (Gıdık 2021).

However, the α‐linolenic acid content of *B. chrysogonum* was 1.07%. Several factors, including genetic variation, plant parts, extraction method, and environmental conditions (e.g., climate, light intensity, temperature, soil composition), can affect the fatty acid composition in BCEO (Mugao 2024; Tomé‐Rodríguez et al. 2023).

### 3.2 Tocols Profile

Due to the antioxidant potential of tocopherols and tocotrienols, determining these bioactive compounds in BCEO is crucial. According to the HPLC results (Table 2), γ‐tocotrienols (794.29 μg/g), *α*‐tocotrienols (635.88 μg/g), and *α*‐tocopherol (540.85 μg/g) were the predominant tocopherols and tocotrienols in BCEO. β‐ and δ‐tocopherol/tocotrienols were also detected, although their concentrations were lower compared to *α* and γ‐tocopherol and tocotrienols. Blueberry seed oil was similarly rich in γ‐tocotrienol (1244.78 mg/kg of oil), but its tocopherol content was comparatively low (Li et al. 2016). In grape seed oil, α‐tocopherol was also the most abundant tocopherol, followed by γ‐tocopherol (Carmona‐Jiménez et al. 2022). Meanwhile, α‐tocopherol (94.0 mg/100 g) and γ‐tocopherol (17.1 mg/100 g) were the primary tocopherols identified in *Berberis integerrima* seed oil (Atefeh Tavakoli et al. 2017).

**TABLE 2:** Profiles of tocopherols of *B. chrysogonum* seed essential oil.

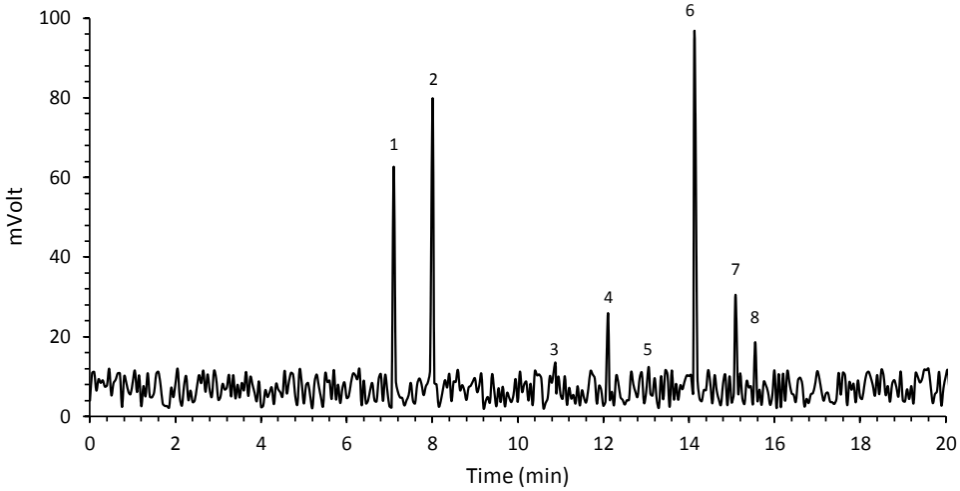

Peak #	Chemical	RT (min)	Concentration (μg/g)
**1**	**α‐Tocopherol (α‐Toc)**	**7.1**	**540.85**
**2**	**α‐Tocotrienols (α‐T3)**	**8.01**	**635.88**
3	β ‐Tocopherol (β‐Toc)	10.88	29.95
4	γ‐ Tocopherol (γ‐Toc)	12.11	99.43
5	β ‐Tocotrienols (β‐T3)	13.06	29.70
**6**	**γ ‐Tocotrienols (γ‐T3)**	**14.13**	**794.29**
7	δ‐Tocopherol (δ‐Toc)	15.09	112.17
8	δ‐Tocotrienols (δ‐T3)	15.55	61.05

*Note:* The bold rows indicate the main components of the essential oil.

Abbreviation: RT, retention time.

### 3.3 Phenolic Compounds Profile

Table 3 presents the phenolic compounds profile of *B. chrysogonum* seed essential oil. The major phenolic compounds identified were quercetin (28.06 μg/mg), luteolin (22.04 μg/mg), rutin (17.43 μg/mg), quercitrin (11.60 μg/mg), P‐OH‐Benzoic acid (10.03 μg/mg), caffeic acid (8.18 μg/mg), m‐Coumaric acid (7.97 μg/mg), myricetin (7.78 μg/mg), and kaempferol (7.59 μg/mg).

**TABLE 3:** Profiles of phenolic compounds of *B. chrysogonum* seed essential oil.

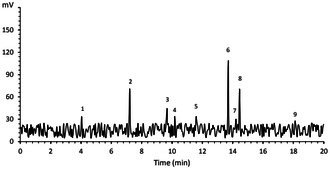

Peak #	Chemical	RT (min)	Concentration (μg/mg)
1	P‐OH‐Benzoic acid	4.04	10.03
2	Rutin	7.21	17.43
3	Quercitrin	9.67	11.60
4	m‐Coumaric acid	10.18	7.97
5	Myricetin	11.59	7.78
**5**	**Quercetin**	**13.71**	**28.06**
6	Caffeic acid	14.22	8.18
7	Luteolin	14.45	22.04
9	Kaempferol	18.11	7.59

*Note:* The bold rows indicate the main components of the essential oil.

Abbreviation: RT, retention time.

Various phenolic compounds, including chlorogenic acid, rutin hydrate, quinic acid, procyanidin, and caffeic acid, were detected in the fruit extract of Berberis crataegina (Ercan 2024). Studies have reported that the rutin content in *Berberis vulgaris* fruit extracts obtained through supercritical carbon dioxide and the Soxhlet method was 173 ± 14.97 μg/g and 208.81 ± 8.48 μg/g, respectively (Nuralın and Gürü 2022). However, catechin (130.29 ppm) was identified as the major component in the seeds of *Berberis vulgaris* (Iskender et al. 2024). Additionally, rutin and quercetin have been reported in grape seed essential oils and extracts (Lingua et al. 2016; Rockenbach et al. 2011; Szabó et al. 2021). Therefore, the investigation of the chemical composition of *B. chrysogonum* seed essential oil showed that this extract contains a wide variety of bioactive compounds. These compounds may be responsible for the antioxidant and antimicrobial potential of this essential oil.

### 3.4 The Total Phenolic Content (TPC)

The TPC of BCEO was 34.75 mg GAE/100 g, which is comparable to that of those for *Barberry Integerrima* seed oil (30.23 mg GAE/100 g), grape seed oil (10.07–136.07 mg GAE/100 g), olive oil (30–50 mg GAE/100 g; Atefe Tavakoli et al. 2016), and sunflower seed oil (49.53 mg GAE/100 g; Kumar et al. 2025). Konuskan et al. (2019) reported TPC values in grape seed oil ranging from 10.2 mg GAE/100 g (Sauvignon Blanc variety and cold‐press extraction method) to 45.2 mg GAE/100 g (Cabernet Sauvignon variety, solvent extraction method). The TPC of BCEO was higher than those reported for various vegetable oils such as hemp (2.45 mg GAE/100 g), flax seed (19.64 mg GAE/100 g; Herchi et al. 2011), and grape seed (10.07 mg GAE/100 g; de Souza et al. 2020).

### 3.5 Oxidizability Value (COX)

The COX value is a good parameter used to assess an oil's tendency to autoxidize. The COX values of BCEO were 5.58, comparable to sunflower oil (5.65) and sesame oil (4.47) (Ghosh et al. 2019) and higher than peanut oil (4.63) (Symoniuk et al. 2022). This value is significantly greater than those reported for palm oil (1.62), camelina oil (1.77), and virgin olive oil (2.37). These results suggest that BCEO may undergo oxidation more rapidly compared to oils with lower COX values. The rate of oxidation depends on various factors such as the degree of unsaturation, the presence of natural antioxidants, and the prior storage conditions (Symoniuk et al. 2022).

### 3.6 The Effect of Essential Oil on Oxidative Stability of Butter

**FIGURE 1:**
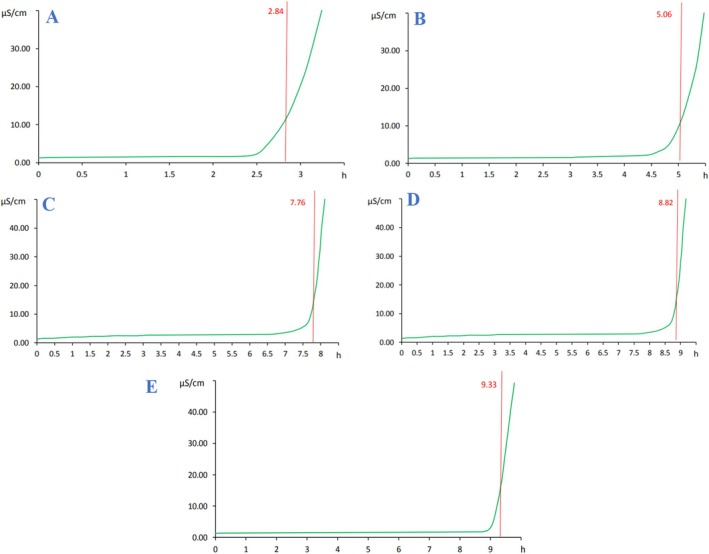
Oxidative induction time (OIT) values for (A) Control butter sample; (B) butter sample containing 250 ppm of *B. chrysogonum* seed essential oil (BCEO); (C) butter sample containing 500 ppm of BCEO; (D) butter sample containing 1000 ppm BCEO; (E) butter sample containing 100 ppm of BHA; BCEO, *B. chrysogonum* seed essential oil.

The oxidative stability was assessed using the Rancimat method, which offers insights into the resistance of oil samples to oxidation under accelerated conditions. According to Figure 1, the oxidative induction time (OIT) value for the butter sample was 2.84 h (Figure 1A). The incorporation of 250 ppm of *B. chrysogonum* seed essential oil into butter increased this time to 5.06 h (Figure 1B). Further elevating the concentration of BCEO to 1000 ppm extended this duration to around 9 h, a value comparable to that obtained for butter containing BHA (9.33 h). In this regard, Golmakani et al. (2021) reported that clove essential oils were effectively enhancing the oxidative induction time of Kilka oil. Additionally, it was demonstrated that the incorporation of essential oils (e.g., rosemary, pennyroyal, thyme, mint, peppermint, mastic gum, and frankincense) was more effective than TBHQ in improving the oxidative stability of black seed oil (Soltani et al. 2024). The high tocopherol content and the presence of various bioactive compounds, such as phenolics and terpenes with antioxidant potential in BCEO, have contributed to the improved oxidative stability of butter containing *B. chrysogonum* seed essential oil (Ciemniewska‐Żytkiewicz et al. 2014).

### 3.7 FTIR Analysis of Encapsulated Samples

**FIGURE 2:**
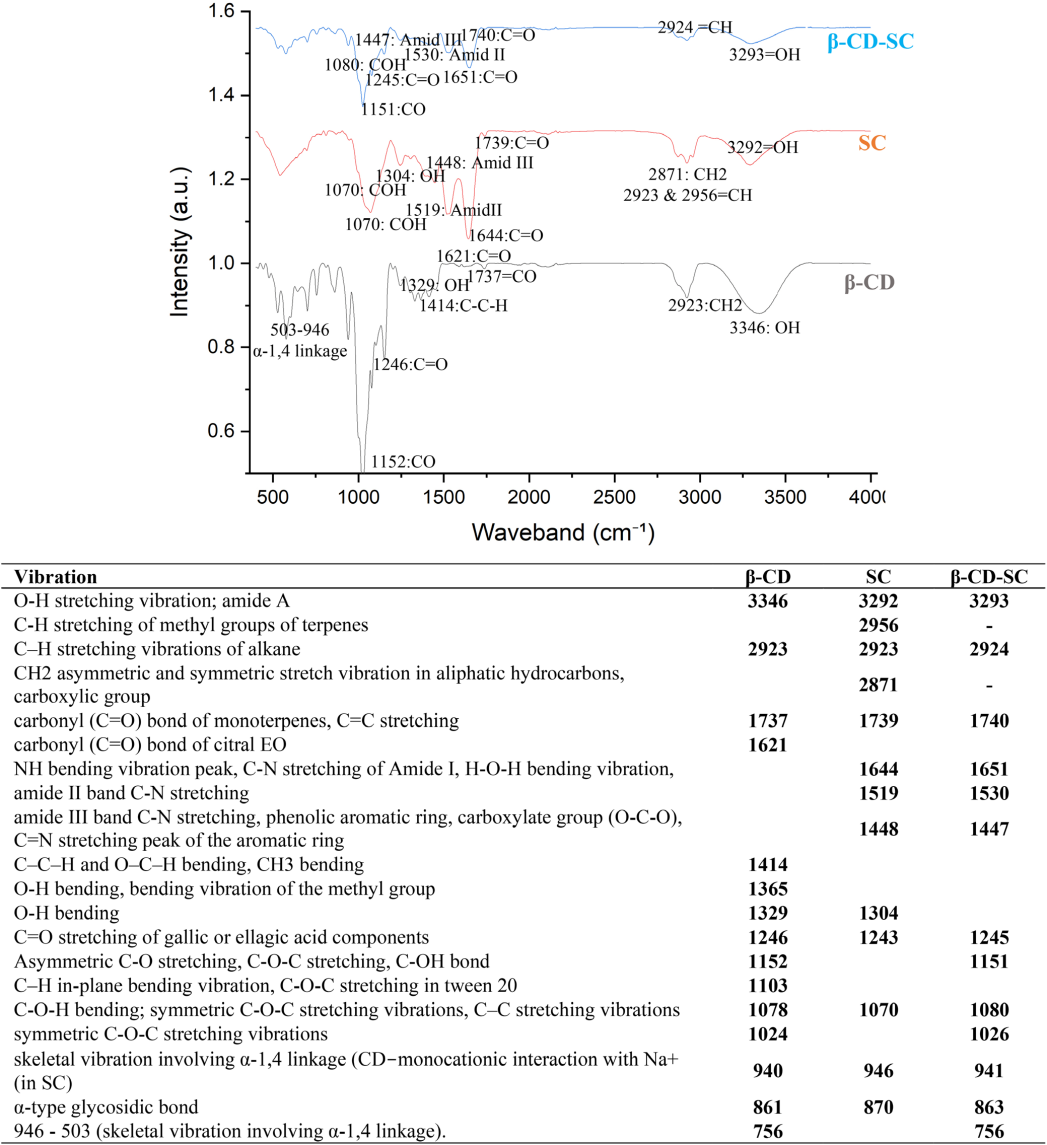
FTIR spectrum of *B. chrysogonum* seed essential oil encapsulated into β‐cyclodextrin (β‐CD), sodium caseinate (SC), and a combination of β‐cyclodextrin and sodium caseinate (β‐CD‐SC).

FTIR analysis is a robust method for identifying structural changes and the development of chemical complexes, as indicated by shifts in the appearance or disappearance of and variations in the intensity of absorption bands caused by inter‐ or intra‐molecular interactions (Magri et al. 2025). The FTIR spectra of β‐CD, SC, and β‐CD‐SC particles are presented in Figure 2. These spectra demonstrate two key peaks: O‐H stretching vibrations occurring around 3200–3500 cm^−1^, which reflect strong inter‐ and intra‐molecular hydrogen bonds, and C‐H stretching at 2969 cm^−1^, which indicates hydrophobic characteristics (Cheong et al. 2018).

The incorporation of β‐CD into SC modified the intensity of amide A and induced peak shifts in the 2800–3300 cm^−1^ range. This shift suggests hydrogen bonding and alkyl C‐H interactions between β‐CD and SC (Cheong et al. 2018). The FTIR spectrum of SC displays distinct amide peaks, including the amide I band at 1644 cm^−1^, the amide II band at 1519 cm^−1^, and the amide III band at 1448 cm^−1^ (Fu et al. 2024). The amide I band is a prominent feature in protein FTIR spectra (i.e., SC sample), primarily involving C‐O stretching of peptide groups and providing insights into the secondary structure of proteins (Xu et al. 2024). The addition of β‐CD resulted in a shift of the amide I band in SC, indicating hydrophobic interactions between β‐CD and SC.

The peaks at 1738 cm^−1^, 2956 cm^−1^, and 2871 cm^−1^ correspond to the carbonyl (C=O) bond of monoterpenes and C‐H stretching of methyl groups in terpenes and alkanes (Magri et al. 2025). The region between 400 and 1500 cm^−1^ is attributed to ‐CH₃ stretching of terpenes and in‐plane C‐H bending vibrations. Additionally, the peak at 1240 cm^−1^ corresponds to C=O stretching of gallic or ellagic acid components, while the band at 860 cm^−1^ arises from C‐H out‐of‐plane bending vibrations of aromatic compounds (Escobar‐Avello et al. 2021). The reduction in intensity of these peaks indicates the successful encapsulation of essential oil components within the microcapsules (Cheong et al. 2018). The disappearance of certain peaks associated with bioactive compounds of the essential oil in β‐CD is likely due to their incorporation within the β‐CD cavities and being overshadowed by the spectral contributions of β‐CD (Escobar‐Avello et al. 2021; Magri et al. 2025).

In the β‐CD spectrum, peaks at 1152 cm^−1^, 1078 cm^−1^, and 1024 cm^−1^ were attributed to C‐O‐C stretching vibrations of sugar molecules. These peaks were also present in the β‐CD‐SC inclusion complex but exhibited slight shifts, providing compelling evidence of successful interactions between SC and β‐CD (Fu et al. 2024). The shift of the amide I band from 1644 cm^−1^ in SC to 1651 cm^−1^ in β‐CD‐SC suggests the formation of hydrogen bonds between BCEO functional groups and encapsulating agents, indicating successful molecular interaction during complex formation (Williams et al. 2021). Overall, significant variations in peak intensity, along with the disappearance and shifting of specific bands in the β‐CD‐SC sample, confirm the formation of a complex between β‐CD and SC, as well as the successful encapsulation of the BCEO.

### 3.8 SEM Images of Encapsulated Samples

**FIGURE 3:**
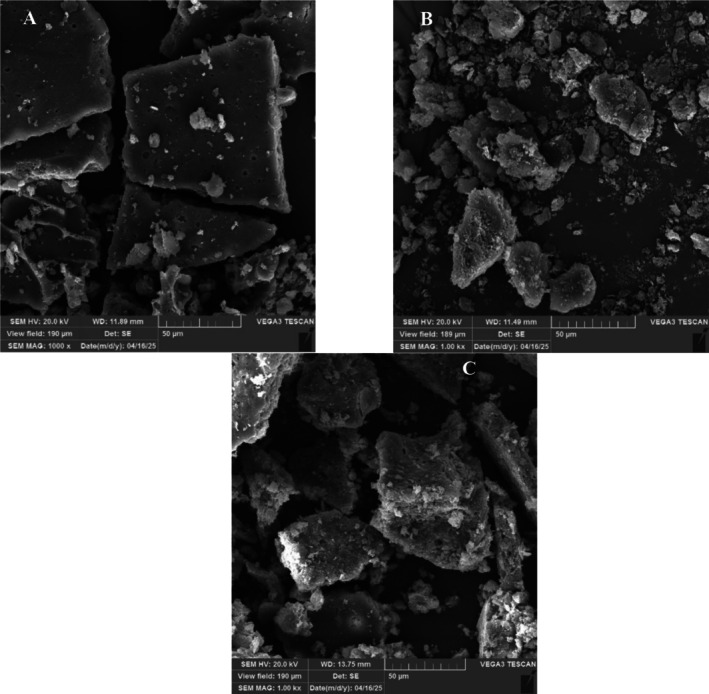
Scanning Electron Microscopy (SEM) images of *B. chrysogonum* seed essential oil encapsulated in β‐cyclodextrin (β‐CD), sodium caseinate (SC), and a combination of β‐cyclodextrin and sodium caseinate (β‐CD‐SC).

Figure 3 presents the surface morphology of β‐CD, SC, and β‐CD‐SC microparticles. The freeze‐dried particles exhibited distinct morphological features, which were influenced by the type of wall material used in their formulation. All samples displayed irregular, glass‐like shapes with uneven and brittle, flaky, porous, and crystalline structures. A similar morphology has been previously reported for freeze‐dried particles (Hundre et al. 2015; Pudziuvelyte et al. 2020; Zhou et al. 2025). Variations in particle size may be related to differences in wall material composition as well as mechanical breakage and grinding intensity during powder handling after freeze‐drying (Pudziuvelyte et al. 2020). Additionally, the presence of fine pores on the surfaces of the particles is likely due to the sublimation of ice crystals during the drying process (Isleroglu et al. 2019).

As shown in Figure 3A, SC particles exhibited an irregular block‐like structure with low agglomeration and larger dimensions compared to other samples. The relatively large particle size of SC can be attributed to the inherently larger molecular size of proteins compared to carbohydrates (Senanayaka 2006). Additionally, SC particles had a smoother surface than the other samples, a finding consistent with Hundre et al. (2015).

In contrast, β‐CD particles (Figure 3B) showed the highest degree of crystal agglomeration. Additionally, the microparticle typically consisted of crystals of varying sizes, exhibiting irregular, rhomboidal‐rectangular shapes and rough surfaces. Among all samples, β‐CD produced the smallest and most irregularly shaped particles. A similar pattern was observed in previous studies (Anaya‐Castro et al. 2017; Magri et al. 2025; Oliveira et al. 2019). The agglomerated structure observed in both β‐CD and β‐CD‐SC samples has been associated with the glass transition of amorphous carbohydrate matrices (Hundre et al. 2015). Such agglomeration may enhance the protection of encapsulated materials (i.e., BCEO) by reducing the exposed surface area of the powder and limiting its contact with environmental factors such as oxygen (Long et al. 2024). These findings were in agreement with the entrapment efficiency data, where β‐CD‐SC and β‐CD samples exhibited the highest EE value.

The β‐CD‐SC sample (Figure 3C) also showed a rough surface and lamellar shape morphology. These particles displayed a flaky structure with multiple lamellar crystals on the surface, as previously described by Acar et al. (2024). Similar structural characteristics have also been reported for freeze‐dried sodium caseinate–carrageenan complex‐loaded β‐carotene powders (Long et al. 2024). These findings suggest that the complexation of SC and β‐CD through intermolecular forces leads to the formation of a lamellar and porous structure at low temperatures during freezing. It has been suggested that such porosity may indicate electrostatic interactions between SC and β‐CD (Jamshidian and Rafe 2024). The interaction between protein and polysaccharide involved multiple factors that altered their spatial structure (Xu et al. 2024).

### 3.9 Entrapment Efficiency of Encapsulated Samples

The entrapment efficiency (EE%) of *B. chrysogonum* seed essential oil in various wall materials was presented in Table 4. The results showed that the EE of BCEO was significantly higher when encapsulated in β‐CD (83.37%) compared to SC (7.24%). The highest EE% was achieved using the β‐CD–SC composite system (88.48%), and this value was significantly different from those of the individual wall materials (*p* < 0.05). This is consistent with previously reported values for Kenaf seed oil encapsulated in gum Arabic, β‐cyclodextrin, and sodium caseinate (Chew et al. 2018) as well as Bergamot essential oil encapsulated in β‐cyclodextrin (~90%) (Maraulo et al. 2024). The superior entrapment potential of β‐CD can be attributed to its amphiphilic structure, characterized by a hydrophilic outer surface and a hydrophobic inner cavity. This unique configuration facilitates the incorporation of non‐polar compounds such as essential oils, through hydrophobic interactions, while the hydrophilic exterior enhances solubility and interfacial stability (Ez‐zoubi et al. 2024; Kringel et al. 2021). Moreover, the enhanced EE% observed in the β‐CD–SC system is likely due to synergistic interactions between the polysaccharide (β‐CD) and the protein (SC) (Cortés‐Morales et al. 2021; Khoshnoudi‐Nia et al. 2022). SC, being a flexible protein with multiple hydrophobic domains, can penetrate the β‐CD cavity and form a viscoelastic network around the essential oil droplets, stabilizing them through various adsorption sites (Xu et al. 2024). This observation supports the findings of Fu et al. (2024) who reported that glycosylated NaCas‐β‐CD conjugates (EE: 96.8%) exhibited enhanced surface hydrophobicity, leading to stronger hydrophobic interactions between the protein and resveratrol (EE: 96.8%), compared to NaCas alone (89.7%) (Fu et al. 2024).

**TABLE 4:** Entrapment Efficiency (EE) and physical properties of *B. chrysogonum* seed essential oil encapsulated into β‐cyclodextrin (β‐CD), sodium caseinate (SC), and a combination of β‐cyclodextrin and sodium caseinate (β‐CD‐SC).

Parameters	β‐CD	SC	β‐CD‐SC	*p*
EE (%)	83.37 ± 0.42^B^	77.24 ± 0.90^C^	88.48 ± 0.57^A^	< 0.0001**
DPPH (%)	84.81 ± 0.87^B^	80.14 ± 0.79^C^	87.59 ± 0.10^A^	< 0.0001**
Moisture content (%)	3.62 ± 0.16^B^	4.38 ± 0.21^A^	3.88 ± 0.15^B^	0.004**
Bulk density (kg/m^3^)	0.584 ± 0.01^A^	0.579 ± 0.01^C^	0.582 ± 0.02^B^	< 0.0001**
Particle size (nm)	179.6 ± 24.43^C^	213.9 ± 21.15^A^	206.4 ± 25.26^B^	< 0.0001**

*Note:* Data are represented as mean ± SD (*n* = 3). Different superscripts in the same row correspond to a significant difference between various samples (*p <* 0.05).

Abbreviations: DPPH, DPPH scavenging activity; EE, entrapment efficiency; PDI, Particle Distribution Index.

** indicate significant effect of wall material on chemical and physical variables at the 1% level (*p* < 0.01).

### 3.10 DPPH Scavenging Potential of Encapsulated Samples

The antioxidant properties of the encapsulated samples were assessed based on their DPPH radical scavenging activities (Table 4). The scavenging activity ranged from 80.14% for SC to 87.59% for β‐CD‐SC, indicating the strong antioxidant potential of these particles. The high antioxidant activity of the encapsulated *B. chrysogonum* seed essential oil may be attributed to the presence of bioactive compounds such as tocopherols, phenolic substances, and terpenes (Hoseini et al. 2025). These bioactive compounds act as antioxidants primarily by neutralizing free radicals through electron donation or reduction reactions, and by chelating pro‐oxidant metal ions (Yadav 2023). These findings are consistent with the entrapment efficiency results, as higher encapsulation efficiency can correspond to a greater concentration of bioactive compounds, thereby enhancing antioxidant capacity (Sharif et al. 2020).

### 3.11 Moisture Content of Encapsulated Samples

As shown in Table 4, the moisture content was significantly higher in the SC‐encapsulated sample (4.38%) compared to the other two microparticles. No significant difference was observed between the moisture levels of β‐CD (3.62%) and β‐CD‐SC (3.88%) at the 5% significance level. The moisture content of all samples was within the acceptable range of 2%–5%, which is considered appropriate for powders intended for food applications. This range helps to minimize the risks associated with microbial growth, lipid oxidation, and caking (Chew et al. 2018; Hoseini et al. 2025). The results were comparable to those reported for kenaf seed oil encapsulated in Gum Arabic, β‐Cyclodextrin, and Sodium Caseinate (2.7%–3.9%) and Fennel essential oil encapsulated in β‐Cyclodextrin‐maltodextrin or gum Arabic (2.76%–6.91%) (Chew et al. 2018; Repajić et al. 2024). The relatively highest moisture content in SC‐based microparticles can be attributed to the hydrophilic nature of sodium caseinate, which facilitates water absorption through hydrogen bonding and capillary attraction between molecular chains (Board 2013; Daniloski et al. 2022). In contrast, the lower moisture content in β‐CD particles is likely due to the hydrophobic nature of the β‐CD cavity (Table 4).

### 3.12 Bulk Density of Encapsulated Samples

Bulk density (BD) is an important physical property that significantly affects the transportation, storage, and packaging of powder materials (Goyal et al. 2015). As presented in Table 4, the bulk density of the samples ranged from 0.579 g/cm^3^ (SC) to 0.584 g/cm^3^ (β‐CD), indicating that the type of wall material significantly influenced the bulk density of the microcapsules. The bulk density is affected by various factors, including the molecular weight and structure of the carrier agent, as well as the particle size and moisture content of the microcapsules (Chew et al. 2018; Shelke et al. 2023). These results are consistent with previous studies on encapsulated essential oils (Balci‐Torun 2024; Hoseini et al. 2025). The smaller particle size of β‐CD reduces interparticle spacing, leading to a higher bulk density. Moreover, SC has a more porous and less dense structure than other samples, which may also explain the lower bulk density of SC particles (ChemicalBook, 2025a, 2025b).

### 3.13 Particle Distribution Index of Encapsulated Samples

**FIGURE 4:**
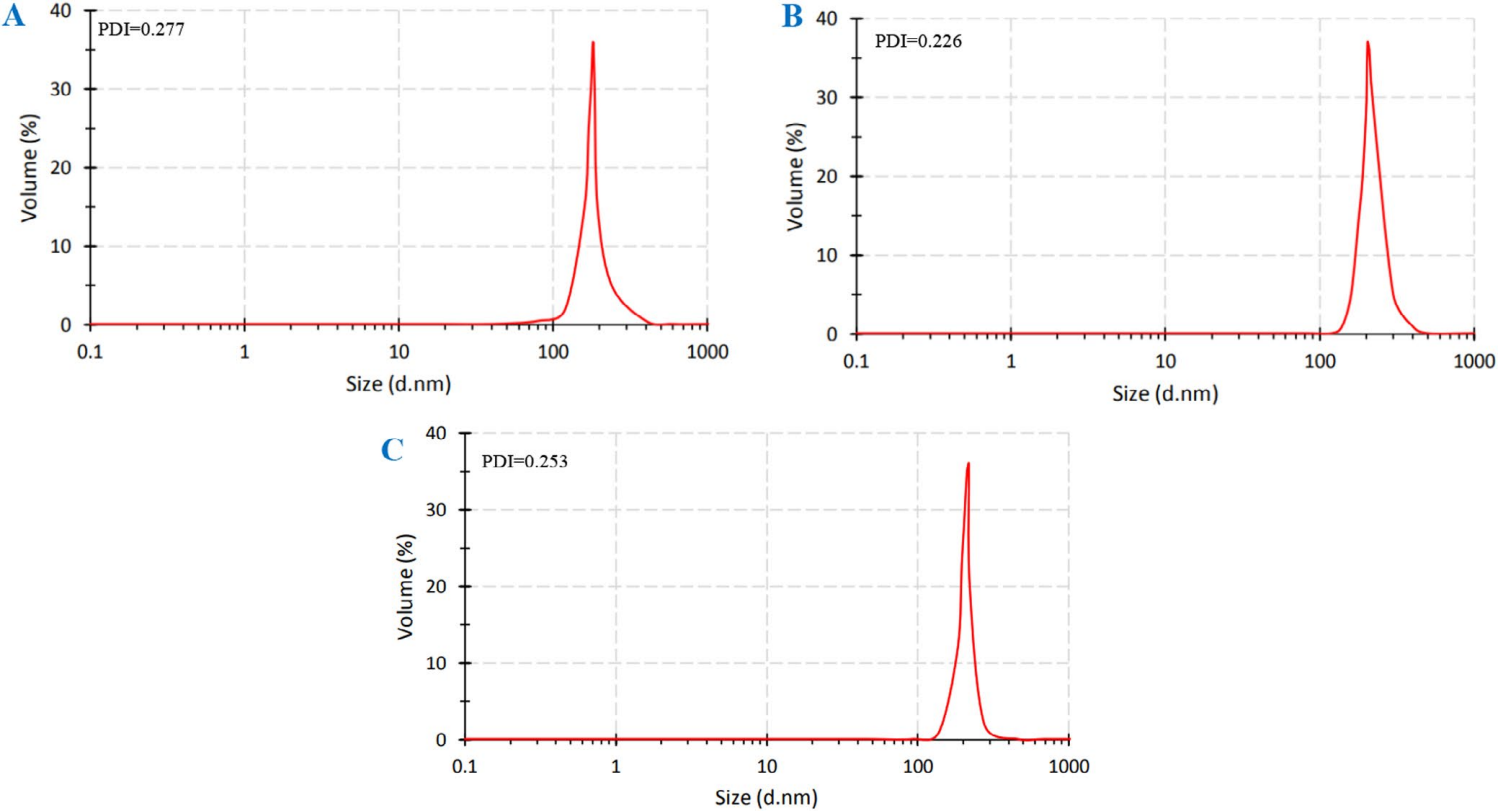
Particle size distribution curve and polydispersity index (PDI) of *B. chrysogonum* seed essential oil encapsulated into (A) β‐cyclodextrin (β‐CD); (B) sodium caseinate (SC); and (C) a combination of β‐cyclodextrin and sodium caseinate (β‐CD‐SC).

As presented in Table 4 and Figure 4, β‐CD particles exhibited a mean particle size of 179.6 ± 24.43 nm, with a PDI of 0.277. The particle size varied from 91.28 nm (0.3%) to 295.3 nm, with over 70% of the particles in the range of 164.2 nm (21.2%) to 190.1 nm (49.6%). The largest particles were observed in the SC formulation, ranging from 141.8 nm (0.4%) to 295.3 nm (1.1%), with a mean size of 213.9 ± 21.15 nm and a PDI of 0.226. Around 71% of these particles were 220.2 nm, and 15.4% were 190.1 nm. The β‐CD‐SC particles demonstrated a narrow particle size distribution, with a PDI of 0.253 and particle sizes ranging from 122.4 nm to 295.3 nm. Notably, 85.5% of the particles were in the size range of 190.1 nm (38.9%) to 220.2 nm (46.6%). The determined range of particle sizes for BCEO powders is consistent with the results of other studies (Chew et al. 2018; Jin and Zhang 2024). Additionally, Repajić et al. (2024) reported that the type of wall material significantly influenced the particle size of encapsulated particles, reporting that the use of β‐cyclodextrin in the particle wall formulation resulted in smaller particle sizes. Encapsulated particles can be classified into three categories based on size: macro‐ (> 5000 μm), micro‐ (0.2–5000 μm), and nano‐particles (< 0.2 μm; Repajić et al. 2024). Consequently, β‐CD can be categorized as nanoparticles. A low PDI value (PDI < 0.3) of all particles reflects a narrow particle size distribution, indicating uniformity and good stability of the samples (Hoseini et al. 2025).

## 4 Conclusions

The findings of this study highlight *Bongardia chrysogonum* seed essential oil (BCEO) as a novel and promising source of natural bioactive compounds with notable antioxidant potential. BCEO was found to be rich in unsaturated fatty acids, tocopherols, tocotrienols, and phenolic compounds such as quercetin, luteolin, and rutin. At a concentration of 1000 ppm, BCEO demonstrated antioxidant efficacy comparable to that of the synthetic antioxidant BHA, and its incorporation into butter extended the oxidative induction time nearly threefold compared to the control sample. Encapsulation of BCEO using β‐cyclodextrin (β‐CD) and sodium caseinate (SC) significantly improved its stability and preserved antioxidant activity. Among the microparticles, the β‐CD–SC composite exhibited the highest entrapment efficiency, favorable particle characteristics, and strong radical scavenging activity, supporting its suitability as a delivery system. Overall, these results support the potential application of BCEO as a natural antioxidant and functional additive in food and nutraceutical products. Importantly, the β‐CD–SC encapsulation method shows potential for scalability, making it a promising candidate for industrial food and nutraceutical applications. It is suggested that future research incorporate complementary antioxidant assays, including ABTS, ORAC, and FRAP, to obtain a broader and more reliable assessment of antioxidant activity. In addition, it should be noted that antioxidant activity was assessed under in vitro conditions, which may not fully represent the complex behavior of antioxidants in real food matrices. Therefore, future studies are encouraged to investigate the release behavior of the encapsulated BCEO under real food processing and storage conditions, assess its effectiveness in preventing oxidative spoilage in various food systems, and further optimize the encapsulation process for large‐scale production.

## Author Contributions

**Mohamad Mehdi Nematshahi:** conceptualization (equal), data curation (equal), formal analysis (equal), investigation (equal), methodology (equal), project administration (equal), supervision (lead), validation (lead), visualization (lead), writing – original draft (equal). **Nafiseh Nemat Shahi:** conceptualization (equal), data curation (equal), formal analysis (equal), investigation (equal), methodology (equal), supervision (equal), validation (equal), visualization (equal). **Rozhan Sanavi Kordestani:** conceptualization (equal), data curation (equal), formal analysis (equal), methodology (equal), writing – original draft (lead).

## Ethics Statement

The authors have nothing to report.

## Conflicts of Interest

The authors declare no conflicts of interest.

## Data Availability

The datasets generated and analyzed during the current study are available from the corresponding author upon reasonable request.

## References

[fsn371085-bib-0001] Abbasi, F. , F. Samadi , S. M. Jafari , S. Ramezanpour , and M. Shams Shargh . 2019. “Ultrasound‐Assisted Preparation of Flaxseed Oil Nanoemulsions Coated With Alginate‐Whey Protein for Targeted Delivery of Omega‐3 Fatty Acids Into the Lower Sections of Gastrointestinal Tract to Enrich Broiler Meat.” Ultrasonics Sonochemistry 50: 208–217. 10.1016/j.ultsonch.2018.09.014. 30249371

[fsn371085-bib-0002] Abdullah, E. C. , and D. Geldart . 1999. “The Use of Bulk Density Measurements as Flowability Indicators.” Powder Technology 102, no. 2: 151–165.

[fsn371085-bib-0003] Abuhamdah, S. , A. Shatarat , M. Al‐Essa , H. Al‐Ameer , S. Al‐Olimat , and P. Chazot . 2017. “Spasmolytic and Antimicrobial Activities of Crude Extract of *Bongardia chrysogonum* L. Tubers.” International Journal of Pharmacology 14, no. 1: 52–60.

[fsn371085-bib-0004] Acar, T. , P. P. Arayici , B. Ucar , et al. 2024. “Host–Guest Interactions of Caffeic Acid Phenethyl Ester With β‐Cyclodextrins: Preparation, Characterization, and In Vitro Antioxidant and Antibacterial Activity.” ACS Omega 9, no. 3: 3625–3634. 38284065 10.1021/acsomega.3c07643 PMC10809231

[fsn371085-bib-0005] Al‐Bukhaiti, W. , A. Noman , A. Ali , S. Abed , and H. Wang . 2018. “Nutritional Composition, Phenolic Compounds Extraction and Antioxidant Activities of Wild Plants: A Review.” American Journal of Food and Nutrition 5: 55–63.

[fsn371085-bib-0006] Alves, E. , A. Simoes , and M. R. Domingues . 2021. “Fruit Seeds and Their Oils as Promising Sources of Value‐Added Lipids From Agro‐Industrial Byproducts: Oil Content, Lipid Composition, Lipid Analysis, Biological Activity and Potential Biotechnological Applications.” Critical Reviews in Food Science and Nutrition 61, no. 8: 1305–1339. 32393054 10.1080/10408398.2020.1757617

[fsn371085-bib-0007] Anaya‐Castro, M. A. , J. F. Ayala‐Zavala , L. Muñoz‐Castellanos , L. Hernández‐Ochoa , J. Peydecastaing , and V. Durrieu . 2017. “β‐Cyclodextrin Inclusion Complexes Containing Clove ( *Eugenia caryophyllata* ) and Mexican Oregano (*Lippia berlandieri*) Essential Oils: Preparation, Physicochemical and Antimicrobial Characterization.” Food Packaging and Shelf Life 14: 96–101.

[fsn371085-bib-0008] AOCS . 2000. Oil Stability Index Sampling and Analysis of Commercial Fats and Oils, 1–2. AOCS Press.

[fsn371085-bib-0009] AOCS . 2024. Tocopherols and Tocotrienols in Vegetable Oils and Fats by HPLC. Vol. Ce 8‐89, 1–10. American Oil Chemists' Society.

[fsn371085-bib-0010] Atanassova, M. , S. Georgieva , and K. Ivancheva . 2011. “Total Phenolic and Total Flavonoid Contents, Antioxidant Capacity and Biological Contaminants in Medicinal Herbs.” Journal of the University of Chemical Technology and Metallurgy 46, no. 1: 81–88.

[fsn371085-bib-0011] Balci‐Torun, F. 2024. “Encapsulation of *Origanum onites* Essential Oil With Different Wall Material Using Spray Drying.” Phytochemical Analysis 35, no. 8: 1736–1747. 10.1002/pca.3218. 36988221

[fsn371085-bib-0012] Board, N. 2013. Confectionery Products Handbook (Chocolate, Toffees, Chewing gum & Sugar Free Confectionery): Food Processing & Agro Based Profitable Projects, Food Processing Industry in India, Bread Manufacturing Project, Candy Manufacturing Process, Food Processing Projects, How to Start a Food Production Business, How to Start Confectionery and Bakery Processing Industry in India, How to Start Food Processing Industry in India. Asia Pacific Business Press Inc.

[fsn371085-bib-0013] Carmona‐Jiménez, Y. , J. M. Igartuburu , D. A. Guillén‐Sánchez , and M. V. García‐Moreno . 2022. “Fatty Acid and Tocopherol Composition of Pomace and Seed Oil From Five Grape Varieties Southern Spain.” Molecules 27, no. 20: 6980. 36296576 10.3390/molecules27206980 PMC9609668

[fsn371085-bib-0014] ChemicalBook . 2025a. “ChemicalBook‐ CAS DataBase List‐ Sodium Caseinate.” https://www.chemicalbook.com/ChemicalProductProperty_EN_CB1766479.htm.

[fsn371085-bib-0015] ChemicalBook . 2025b. “ChemicalBook ‐CAS DataBase List‐β‐Cyclodextrin.” https://www.chemicalbook.com/ChemicalProductProperty_EN_CB1766479.htm.

[fsn371085-bib-0016] Chen, C. 2003. “Evaluation of Air Oven Moisture Content Determination Methods for Rough Rice.” Biosystems Engineering 86, no. 4: 447–457.

[fsn371085-bib-0017] Cheong, A. M. , C. P. Tan , and K. L. Nyam . 2018. “Effect of Emulsification Method and Particle Size on the Rate of in Vivo Oral Bioavailability of Kenaf ( *Hibiscus cannabinus* L.) Seed Oil.” Journal of Food Science 83, no. 7: 1964–1969. 10.1111/1750-3841.14191. 29802733

[fsn371085-bib-0018] Chew, S. C. , C. P. Tan , and K. L. Nyam . 2018. “Effect of Gum Arabic, β‐Cyclodextrin, and Sodium Caseinate as Encapsulating Agent on the Oxidative Stability and Bioactive Compounds of Spray‐Dried Kenaf Seed Oil.” Journal of Food Science 83, no. 9: 2288–2294. 30074623 10.1111/1750-3841.14291

[fsn371085-bib-0019] Chorshanbiev, F. , and E. Berdiev . 2022. “Morphology and Composition of Lipids of Barberry Seeds (Berberis Oblonga Rgl.).” Paper presented at the International Scientific Conference on Agricultural Machinery Industry “Interagromash”.

[fsn371085-bib-0020] Ciemniewska‐Żytkiewicz, H. , K. Ratusz , J. Bryś , M. Reder , and P. Koczoń . 2014. “Determination of the Oxidative Stability of Hazelnut Oils by PDSC and Rancimat Methods.” Journal of Thermal Analysis and Calorimetry 118: 875–881.

[fsn371085-bib-0021] Cortés‐Morales, E. A. , G. Mendez‐Montealvo , and G. Velazquez . 2021. “Interactions of the Molecular Assembly of Polysaccharide‐Protein Systems as Encapsulation Materials. A Review.” Advances in Colloid and Interface Science 295: 102398. 10.1016/j.cis.2021.102398. 33931199

[fsn371085-bib-0022] Daniloski, D. , N. A. McCarthy , M. J. Auldist , and T. Vasiljevic . 2022. “Properties of Sodium Caseinate as Affected by the β‐Casein Phenotypes.” Journal of Colloid and Interface Science 626: 939–950. 10.1016/j.jcis.2022.07.021. 35835044

[fsn371085-bib-0023] de Souza, R. d. C. , B. A. S. Machado , G. d. A. Barreto , I. L. Leal , J. P. d. Anjos , and M. A. Umsza‐Guez . 2020. “Effect of Experimental Parameters on the Extraction of Grape Seed Oil Obtained by Low Pressure and Supercritical Fluid Extraction.” Molecules 25, no. 7: 1634. 32252316 10.3390/molecules25071634 PMC7180707

[fsn371085-bib-0024] Devecioglu, D. , O. Atli , A. C. Karaca , and F. Karbancioglu‐Guler . 2025. “Evaluation of the Antimicrobial Effect of Encapsulated Cumin Seed Essential Oil in Chickpea Protein–Maltodextrin Matrix and Its Potential to Extend the Shelf Life of Meatballs.” Food Science and Technology International 1: 10820132241307715. 10.1016/j.molliq.2022.119379. 39748547

[fsn371085-bib-0025] Dokuyucu, R. , K. H. Gozukara , O. Ozcan , et al. 2016. “The Effect of *Bongardia chrysogonum* on Prostate Tissue in a Rat Model of STZ‐Induced Diabetes.” Springerplus 5: 1322. 27563517 10.1186/s40064-016-2973-z PMC4980850

[fsn371085-bib-0026] Ercan, L. 2024. “Bioactive Components, Antioxidant Capacity, and Antimicrobial Activity of *Berberis crataegina* Fruit.” Pharmacological Research‐Natural Products 2: 100020.

[fsn371085-bib-0027] Escobar‐Avello, D. , J. Avendaño‐Godoy , J. Santos , et al. 2021. “Encapsulation of Phenolic Compounds From a Grape Cane Pilot‐Plant Extract in Hydroxypropyl Beta‐Cyclodextrin and Maltodextrin by Spray Drying.” Antioxidants 10, no. 7: 1130. 34356363 10.3390/antiox10071130 PMC8301162

[fsn371085-bib-0028] Ez‐zoubi, A. , H. Zaroual , Y. E. Zoubi , M. Fadil , and A. Farah . 2024. “Inclusion Complex Essential Oil Into Cyclodextrins and Its Optimization via Experimental Designs: a Review.” Chemical Papers 78, no. 7: 4075–4094. 10.1007/s11696-024-03405-6.

[fsn371085-bib-0029] Fenyvesi, E. , C. Zemlényi , J. Orgoványi , E. Oláh , and L. Szente . 2016. “Can Conversion Mixture Substitute Beta‐Cyclodextrin in Encapsulation of Essential Oils and Their Components?” Journal of Inclusion Phenomena and Macrocyclic Chemistry 86, no. 1: 55–66.

[fsn371085-bib-0030] Freitag, H. 1977. “The Pleniglacial, Late Glacial and Early Postglacial Vegetations of Zeribar and Their Present‐Day Counterparts.” In Palynological investigations in Western Iran, edited by W. Van Zeist and S. Bottema , 87–95. Palaeohistoria.

[fsn371085-bib-0031] Fu, Y. , P. Zhu , H. Lin , et al. 2024. “Encapsulation of Resveratrol in Nanosheet Structured Sodium Caseinate Dialdehyde β‐Cyclodextrin Conjugate With Enhanced Physicochemical Properties.” LWT 211: 116910.

[fsn371085-bib-0032] Gezici, S. , and N. Şekeroğlu . 2021. “ *Bongardia chrysogonum* (L.) Spach as a Potential Medicinal Plant Against Cancer and Alzheimer's Disease Management.” İstanbul Journal of Pharmacy 51, no. 3: 319–325.

[fsn371085-bib-0033] Ghosh, M. , R. Upadhyay , D. K. Mahato , and H. N. Mishra . 2019. “Kinetics of Lipid Oxidation in Omega Fatty Acids Rich Blends of Sunflower and Sesame Oils Using Rancimat.” Food Chemistry 272: 471–477. 30309570 10.1016/j.foodchem.2018.08.072

[fsn371085-bib-0034] Gıdık, B. 2021. “Antioxidant, Antimicrobial Activities and Fatty Acid Compositions of Wild Berberis spp. by Different Techniques Combined With Chemometrics (PCA and HCA).” Molecules 26, no. 24: 7448. 34946529 10.3390/molecules26247448 PMC8704344

[fsn371085-bib-0035] Golmakani, M. , E. Dorostkar , and M. Keramat . 2021. “Common Kilka Oil and Its Primary and Secondary Oxidative Dynamics Stabilized by Different Variants of Clove Essential Oil.” Grasas y Aceites 72, no. 1: e390.

[fsn371085-bib-0036] Gorjian, H. , P. Mihankhah , and N. G. Khaligh . 2022. “Influence of Tween Nature and Type on Physicochemical Properties and Stability of Spearmint Essential Oil ( *Mentha spicata* L.) Stabilized With Basil Seed Mucilage Nanoemulsion.” Journal of Molecular Liquids 359: 119379. 10.1016/j.molliq.2022.119379.

[fsn371085-bib-0037] Görmez, G. 2024. “The Anticancer Potential of Van Lake Basin Plants.” Yüzüncü Yıl Üniversitesi Fen Bilimleri Enstitüsü Dergisi 29, no. 2: 787–797.

[fsn371085-bib-0038] Górska, A. , I. Piasecka , M. Wirkowska‐Wojdyła , et al. 2023. “Berry Seeds—A By‐Product of the Fruit Industry as a Source of Oils With Beneficial Nutritional Characteristics.” Applied Sciences 13, no. 8: 5114.

[fsn371085-bib-0039] Goyal, A. , V. Sharma , M. K. Sihag , et al. 2015. “Development and Physico‐Chemical Characterization of Microencapsulated Flaxseed Oil Powder: A Functional Ingredient for Omega‐3 Fortification.” Powder Technology 286: 527–537.

[fsn371085-bib-0040] Gull, T. , B. Sultana , F. Anwar , W. Nouman , T. Mehmood , and M. Sher . 2018. “Characterization of Phenolics in Different Parts of Selected Capparis Species Harvested in Low and High Rainfall Season.” Journal of Food Measurement and Characterization 12, no. 3: 1539–1547.

[fsn371085-bib-0041] Herchi, W. , F. Sakouhi , D. Arráez‐Román , et al. 2011. “Changes in the Content of Phenolic Compounds in Flaxseed Oil During Development.” Journal of the American Oil Chemists' Society 88: 1135–1142.

[fsn371085-bib-0042] Hoseini, S.‐A. , M. Vazifedoost , B. Hajirostamloo , Z. Didar , and M.‐M. N. Shahi . 2025. “Supercritical Fluid Extraction and Encapsulation of Rivas (*Rheum ribes*) Flower: Principal Component Analysis (PCA).” Heliyon 11: e41746. 39872459 10.1016/j.heliyon.2025.e41746 PMC11770504

[fsn371085-bib-0043] Hu, G. , Y. Lu , and D. Wei . 2005. “Fatty Acid Composition of the Seed Oil of *Allium tuberosum* .” Bioresource Technology 96, no. 14: 1630–1632. 10.1016/j.biortech.2004.11.022. 15936941

[fsn371085-bib-0044] Hundre, S. Y. , P. Karthik , and C. Anandharamakrishnan . 2015. “Effect of Whey Protein Isolate and β‐Cyclodextrin Wall Systems on Stability of Microencapsulated Vanillin by Spray–Freeze Drying Method.” Food Chemistry 174: 16–24. 25529646 10.1016/j.foodchem.2014.11.016

[fsn371085-bib-0045] Iskender, H. , S. Ceylan , E. Dokumacioglu , A. Saral Sariyer , A. Karagoz , and H. Akyildirim Begen . 2024. “Determination of Essential Oil and Phenolic Compounds of *Berberis vulgaris* Grown in Şavşat, Artvin; Revealing Its Antioxidant and Antimicrobial Activities.” Zeitschrift für Naturforschung. C, A Journal of Biosciences 80: 391–400. 40607705 10.1515/znc-2024-0163

[fsn371085-bib-0046] Isleroglu, H. , I. Turker , B. Koc , and M. Tokatli . 2019. “Microencapsulation of Microbial Transglutaminase by Ultrasonic Spray‐Freeze Drying.” Food and Bioprocess Technology 12: 2004–2017.

[fsn371085-bib-0047] Jafari, S. M. , K. Mahdavi‐Khazaei , and A. Hemmati‐Kakhki . 2016. “Microencapsulation of Saffron Petal Anthocyanins With Cress Seed Gum Compared With Arabic Gum Through Freeze Drying.” Carbohydrate Polymers 140: 20–25. 10.1016/j.carbpol.2015.11.079. 26876823

[fsn371085-bib-0048] Jamshidian, H. , and A. Rafe . 2024. “Complex Coacervate of Wheat Germ Protein/High Methoxy Pectin in Encapsulation of d‐Limonene.” Chemical and Biological Technologies in Agriculture 11, no. 1: 60.

[fsn371085-bib-0049] Jin, Y. , and S. Zhang . 2024. “Adenosine Encapsulation and Characterization Through Layer‐By‐Layer Assembly of Hydroxypropyl‐β‐Cyclodextrin and Whey Protein Isolate as Wall Materials.” Molecules 29, no. 9: 2046. 10.3390/molecules29092046. 38731538 PMC11085109

[fsn371085-bib-0050] Khoshnoudi‐Nia, S. , Z. Forghani , and S. M. Jafari . 2022. “A Systematic Review and Meta‐Analysis of Fish Oil Encapsulation Within Different Micro/Nanocarriers.” Critical Reviews in Food Science and Nutrition 62, no. 8: 2061–2082. 10.1080/10408398.2020.1848793. 33207958

[fsn371085-bib-0051] Koleva, I. I. , T. A. Van Beek , J. P. Linssen , A. d. Groot , and L. N. Evstatieva . 2002. “Screening of Plant Extracts for Antioxidant Activity: A Comparative Study on Three Testing Methods.” Phytochemical Analysis: An International Journal of Plant Chemical and Biochemical Techniques 13, no. 1: 8–17. 10.1002/pca.611 11899609

[fsn371085-bib-0052] Konuskan, D. B. , O. Kamiloglu , and O. Demirkeser . 2019. “Fatty Acid Composition, Total Phenolic Content and Antioxidant Activity of Grape Seed Oils Obtained by Cold‐Pressed and Solvent Extraction.” Indian Journal Of Pharmaceutical Education And Research 53: 144–150.

[fsn371085-bib-0053] Kringel, D. H. , G. H. Lang , Á. R. G. Dias , E. A. Gandra , T. K. Valente Gandra , and E. da Rosa Zavareze . 2021. “Impact of Encapsulated Orange Essential Oil With β‐Cyclodextrin on Technological, Digestibility, Sensory Properties of Wheat Cakes as Well as *Aspergillus flavus* Spoilage.” Journal of the Science of Food and Agriculture 101, no. 13: 5599–5607. 33709436 10.1002/jsfa.11211

[fsn371085-bib-0054] Kumar, S. , A. Rai , and K. Prasad . 2025. “Enhancing Sustainability and Quality: A Comparative Study of Sunflower Seed Oil Extraction Methods and Physico‐Chemical Characterization.” Sustainable Chemistry One World 6: 100060.

[fsn371085-bib-0055] Li, Q. , J. Wang , and F. Shahidi . 2016. “Chemical Characteristics of Cold‐Pressed Blackberry, Black Raspberry, and Blueberry Seed Oils and the Role of the Minor Components in Their Oxidative Stability.” Journal of Agricultural and Food Chemistry 64, no. 26: 5410–5416. 27203814 10.1021/acs.jafc.6b01821

[fsn371085-bib-0056] Liao, W. , A. Gharsallaoui , E. Dumas , and A. Elaissari . 2022. “Understanding of the Key Factors Influencing the Properties of Emulsions Stabilized by Sodium Caseinate.” Comprehensive Reviews in Food Science and Food Safety 21, no. 6: 5291–5317. 36301626 10.1111/1541-4337.13062

[fsn371085-bib-0057] Lingua, M. S. , M. P. Fabani , D. A. Wunderlin , and M. V. Baroni . 2016. “From Grape to Wine: Changes in Phenolic Composition and Its Influence on Antioxidant Activity.” Food Chemistry 208: 228–238. 27132844 10.1016/j.foodchem.2016.04.009

[fsn371085-bib-0058] Long, Y. , J. Zhang , D. Li , et al. 2024. “The Characterization and Stability of Powdered Oil Loaded With β‐Carotene Prepared From a Sodium Caseinate–Carrageenan Complex: The Effect of Vacuum Freeze‐Drying and Spray‐Drying.” Food 13, no. 22: 3690. 10.3390/foods13223690 PMC11594183 39594105

[fsn371085-bib-0059] Magri, A. , M. Ramos , C. Mellinas , A. Jiménez , and M. C. Garrigós . 2025. “Encapsulation of Lemongrass Essential Oil in Cyclodextrins and Maltodextrin: Antioxidant, Antimicrobial and Release Studies.” Carbohydrate Polymer Technologies and Applications 10: 100749.

[fsn371085-bib-0060] Maraulo, G. E. , C. dos Santos Ferreira , C. E. Beaufort , M. G. Ugarte , and M. F. Mazzobre . 2024. “Encapsulation of Bergamot Essential Oil Components in β‐Cyclodextrin by Ultrasound‐Assisted co‐Precipitation Method: Optimization, Characterization, and Antibacterial Activity.” Food and Bioprocess Technology 17: 5386–5400. 10.1007/s11947-024-03442-9.

[fsn371085-bib-0061] Matthäus, B. 2008. “Virgin Grape Seed Oil: Is It Really a Nutritional Highlight?” European Journal of Lipid Science and Technology 110, no. 7: 645–650. 10.1002/ejlt.200700276.

[fsn371085-bib-0062] Mazzuca, M. , S. Miscoria , E. Rost , and V. Balzaretti . 2005. “Fatty Acids and Sterols in Seeds From Wild Species of Berberis in Argentine Patagonia.” Paper presented at the Anales de la Asociacion Quimica Argentina.

[fsn371085-bib-0063] Mugao, L. 2024. “Factors Influencing Yield, Chemical Composition and Efficacy of Essential Oils.” International Journal of Multidisciplinary Research and Growth Evaluation 5: 169–178. 10.54660/.IJMRGE.2024.5.4.169-178.

[fsn371085-bib-0064] Nova‐Baza, D. , L. Olivares‐Caro , L. Bustamante , et al. 2022. “Metabolic Profile and Antioxidant Capacity of Five Berberis Leaves Species: A Comprehensive Study to Determine Their Potential as Natural Food or Ingredient.” Food Research International 160: 111642. 10.1016/j.foodres.2022.111642. 36076377

[fsn371085-bib-0065] Nuralın, L. , and M. Gürü . 2022. “ *Berberis vulgaris* Fruit: Determination of Phenolic Compounds in Extracts Obtained by Supercritical CO_2_ and Soxhlet Methods Using HPLC.” Food Analytical Methods 15: 877–889. 34812272 10.1007/s12161-021-02136-8 PMC8598104

[fsn371085-bib-0066] Oliveira, A. P. , A. L. Silva , L. G. Viana , et al. 2019. “β‐Cyclodextrin Complex Improves the Bioavailability and Antitumor Potential of Cirsiliol, a Flavone Isolated From *Leonotis nepetifolia* (Lamiaceae).” Heliyon 5, no. 10: e01692. 31720439 10.1016/j.heliyon.2019.e01692 PMC6838880

[fsn371085-bib-0067] Pieroni, A. , H. M. Ahmed , and H. Zahir . 2017. “The Spring Has Arrived: Traditional Wild Vegetables Gathered by Yarsanis (Ahl‐e Haqq) and Sunni Muslims in Western Hawraman, SE Kurdistan (Iraq).” Acta Societatis Botanicorum Poloniae 86, no. 1: 1–17.

[fsn371085-bib-0068] Pudziuvelyte, L. , M. Marksa , K. Sosnowska , K. Winnicka , R. Morkuniene , and J. Bernatoniene . 2020. “Freeze‐Drying Technique for Microencapsulation of *Elsholtzia ciliata* Ethanolic Extract Using Different Coating Materials.” Molecules 25, no. 9: 2237. 32397476 10.3390/molecules25092237 PMC7248874

[fsn371085-bib-0069] Repajić, M. , I. Elez Garofulić , N. Marčac Duraković , et al. 2024. “Physico‐Chemical Characterization of Encapsulated Fennel Essential Oil Under the Influence of Spray‐Drying Conditions.” PRO 12, no. 3: 577. 10.3390/pr12030577.

[fsn371085-bib-0070] Rockenbach, I. I. , L. V. Gonzaga , V. M. Rizelio , A. E. d. S. S. Gonçalves , M. I. Genovese , and R. Fett . 2011. “Phenolic Compounds and Antioxidant Activity of Seed and Skin Extracts of Red Grape (*Vitis vinifera* and *Vitis labrusca*) Pomace From Brazilian Winemaking.” Food Research International 44, no. 4: 897–901.

[fsn371085-bib-0071] Senanayaka, S. K. 2006. Formulation and Development of a Cereal Based Infant Food. (Bachelor of Science in Food Sciences and Technology). Subaragamuwa University Buttala.

[fsn371085-bib-0072] Setyawan, H. , S. Sukardi , and C. Puriwangi . 2021. “Phytochemicals Properties of Avocado Seed: A Review.” Paper presented at the IOP Conference Series: Earth and Environmental Science.

[fsn371085-bib-0073] Sharif, N. , S. Khoshnoudi‐Nia , and S. M. Jafari . 2020. “Nano/Microencapsulation of Anthocyanins; a Systematic Review and Meta‐Analysis.” Food Research International 132: 109077. 10.1016/j.foodres.2020.109077. 32331692

[fsn371085-bib-0074] Shelke, G. , V. Kad , R. Pandiselvam , et al. 2023. “Physical and Functional Stability of Spray‐Dried Jamun ( *Syzygium cumini* L.) Juice Powder Produced With Different Carrier Agents.” Journal of Texture Studies 54: 560–570. 36883842 10.1111/jtxs.12749

[fsn371085-bib-0075] Soltani, R. , S. Azadmard‐Damirchi , M. Gharekhani , and M. Torbati . 2024. “Effect of Some Herbal Essential Oils on the Content of Compounds With Pharmacological Properties and Oxidative Stability of Black Cumin Seed Oil.” Crescent Journal of Medical and Biological Sciences 12, no. 1: 1–17. 10.34172/cjmb.2024.3003.

[fsn371085-bib-0076] Symoniuk, E. , N. Ksibi , M. Wroniak , M. Lefek , and K. Ratusz . 2022. “Oxidative Stability Analysis of Selected Oils From Unconventional Raw Materials Using Rancimat Apparatus.” Applied Sciences 12, no. 20: 10355.

[fsn371085-bib-0077] Szabó, É. , T. Marosvölgyi , G. Szilágyi , et al. 2021. “Correlations Between Total Antioxidant Capacity, Polyphenol and Fatty Acid Content of Native Grape Seed and Pomace of Four Different Grape Varieties in Hungary.” Antioxidants 10, no. 7: 1101. 34356334 10.3390/antiox10071101 PMC8300998

[fsn371085-bib-0078] Tavakoli, A. , M. A. Sahari , and M. Barzegar . 2017. “Antioxidant Activity of *Berberis integerrima* Seed Oil as a Natural Antioxidant on the Oxidative Stability of Soybean Oil.” International Journal of Food Properties 20, no. sup3: S2914–S2925.

[fsn371085-bib-0079] Tavakoli, A. , M. A. Sahari , M. Barzegar , and M. A. Ghajari . 2016. “Physicochemical and Fatty Acids Composition of Barberry Integerrima Seed.” International Journal of Nutrition 1, no. 4: 8–21.

[fsn371085-bib-0080] Tomé‐Rodríguez, S. , F. Barba‐Palomeque , C. Ledesma‐Escobar , H. Miho , C. Díez , and F. Priego‐Capote . 2023. “Influence of Genetic and Interannual Factors on the Fatty Acids Profile of Virgin Olive Oil.” Food Chemistry 422: 136175. 10.1016/j.foodchem.2023.136175. 37116272

[fsn371085-bib-0081] Williams, A. E. , N. I. Hammer , R. C. Fortenberry , and D. N. Reinemann . 2021. “Tracking the Amide i and αcoo− Terminal ν (C= O) Raman Bands in a Family of l‐Glutamic Acid‐Containing Peptide Fragments: A Raman and Dft Study.” Molecules 26, no. 16: 4790. 34443382 10.3390/molecules26164790 PMC8399447

[fsn371085-bib-0082] Xu, X. , Y. Dong , C. Jiang , Z. Zheng , and Z. Dai . 2024. “Study on the Synergistic Action of β‐Cyclodextrin and Sodium Caseinate to Stabilize the Seal Oil Pickering Emulsion.” LWT 205: 116460.

[fsn371085-bib-0083] Yadav, A. S. 2023. “Collating Antioxidant, Reducing and Metal Chelating Properties of Spices and Acacia.” Food Chemistry Advances 2: 100257. 10.1016/j.focha.2023.100257.

[fsn371085-bib-0084] Yang, C. , K. Shang , C. Lin , et al. 2021. “Processing Technologies, Phytochemical Constituents, and Biological Activities of Grape Seed Oil (GSO): A Review.” Trends in Food Science and Technology 116: 1074–1083.

[fsn371085-bib-0085] Yousef, I. , S. Oran , M. Alqaraleh , and Y. Bustanji . 2021. “Evaluation of Cytotoxic, Antioxidant and Antibacterial Activities of Origanum dayi, Salvia palaestina and *Bongardia chrysogonum* Plants Growing Wild in Jordan.” Tropical Journal of Natural Product Research (TJNPR) 5, no. 1: 66–70.

[fsn371085-bib-0086] Zhou, S. , X. Zhang , J. Zhang , et al. 2025. “Differences in Physicochemical Properties and Proteomics Analysis of Spray‐and Freeze‐Dried Milk Powders From Bovine, Goat, and Horse Sources.” Journal of Dairy Science 108, no. 2: 1367–1379. 39521428 10.3168/jds.2024-25146

[fsn371085-bib-0087] Zoubiri, S. , and A. Baaliouamer . 2010. “Essential Oil Composition of *Coriandrum sativum* Seed Cultivated in Algeria as Food Grains Protectant.” Food Chemistry 122, no. 4: 1226–1228. 10.1016/j.foodchem.2010.03.119.

